# Ethyl 2-(4-bromo­phen­yl)-1-phenyl-1*H*-benzimidazole-5-carboxyl­ate

**DOI:** 10.1107/S1600536812019897

**Published:** 2012-05-12

**Authors:** Yeong Keng Yoon, Mohamed Ashraf Ali, Tan Soo Choon, Suhana Arshad, Ibrahim Abdul Razak

**Affiliations:** a Institute for Research in Molecular Medicine, Universiti Sains Malaysia, Minden 11800, Penang, Malaysia; b School of Physics, Universiti Sains Malaysia, 11800 USM, Penang, Malaysia

## Abstract

In the title compound, C_22_H_17_BrN_2_O_2_, the benzimidazole ring system is essentially planar, with a maximum deviation of 0.017 (1) Å, and forms dihedral angles of 27.79 (6) and 64.43 (6)° with the phenyl and bromo-substituted benzene rings, respectively. In the crystal, mol­ecules are linked into one-dimensional chains along the *a* axis by weak C—H⋯O hydrogen bonds. Weak inter­molecular C—H⋯π inter­actions are also present.

## Related literature

For background to and the biological activities of benzimidazoles, see: Townsend & Revankar (1970 ▶); Rao *et al.* (2002 ▶); Thakurdesai *et al.* (2007 ▶); Dubey & Sanyal (2010 ▶); Lacey (1990 ▶). For a related structure, see: Arumugam *et al.* (2010 ▶). For the stability of the temperature controller used for the data collection, see: Cosier & Glazer (1986 ▶).
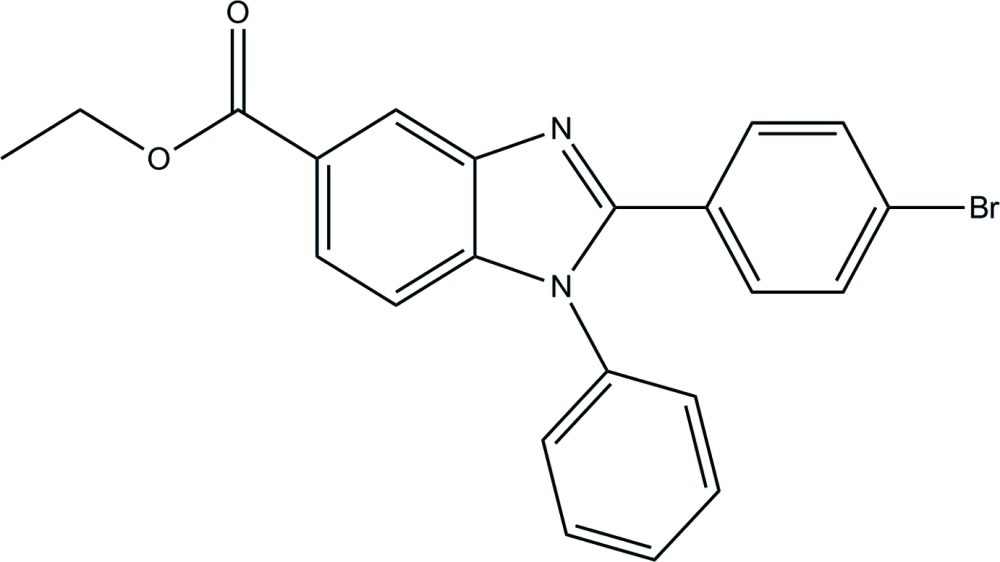


## Experimental

### 

#### Crystal data

C_22_H_17_BrN_2_O_2_
*M* *_r_* = 421.29
Triclinic, 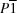

*a* = 9.3121 (2) Å
*b* = 9.8136 (2) Å
*c* = 11.8458 (2) Å
α = 108.217 (1)°
β = 101.135 (1)°
γ = 109.361 (1)°
*V* = 915.39 (3) Å^3^
*Z* = 2
Mo *K*α radiation
μ = 2.27 mm^−1^
*T* = 100 K
0.42 × 0.33 × 0.15 mm

#### Data collection

Bruker SMART APEXII CCD area-detector diffractometer
Absorption correction: multi-scan (*SADABS*; Bruker, 2009 ▶) *T*_min_ = 0.450, *T*_max_ = 0.728
21718 measured reflections
5326 independent reflections
4846 reflections with *I* > 2σ(*I*)
*R*_int_ = 0.025

#### Refinement

*R*[*F* ^2^ > 2σ(*F* ^2^)] = 0.027
*wR*(*F* ^2^) = 0.071
*S* = 1.06
5326 reflections
245 parameters
H-atom parameters constrained
Δρ_max_ = 0.53 e Å^−3^
Δρ_min_ = −0.65 e Å^−3^

### 

Data collection: *APEX2* (Bruker, 2009 ▶); cell refinement: *SAINT* (Bruker, 2009 ▶); data reduction: *SAINT*; program(s) used to solve structure: *SHELXTL* (Sheldrick, 2008 ▶); program(s) used to refine structure: *SHELXTL*; molecular graphics: *SHELXTL*; software used to prepare material for publication: *SHELXTL* and *PLATON* (Spek, 2009 ▶).

## Supplementary Material

Crystal structure: contains datablock(s) global, I. DOI: 10.1107/S1600536812019897/lh5466sup1.cif

Structure factors: contains datablock(s) I. DOI: 10.1107/S1600536812019897/lh5466Isup2.hkl

Supplementary material file. DOI: 10.1107/S1600536812019897/lh5466Isup3.cml

Additional supplementary materials:  crystallographic information; 3D view; checkCIF report

## Figures and Tables

**Table 1:** Hydrogen-bond geometry (Å, °) *Cg*1 and *Cg*2 are the centroids of the N1/N1/C1/C6/C7 and C8–C13 rings, respectively.

*D*—H⋯*A*	*D*—H	H⋯*A*	*D*⋯*A*	*D*—H⋯*A*
C15—H15*A*⋯O2^i^	0.95	2.38	3.311 (2)	166
C19—H19*A*⋯*Cg*2^ii^	0.95	2.59	3.4534 (18)	152
C21—H21*A*⋯*Cg*1^iii^	0.99	2.64	3.5288 (15)	149

Symmetry codes: (i) 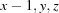; (ii) 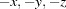; (iii) 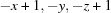.

**Fig. 1.**
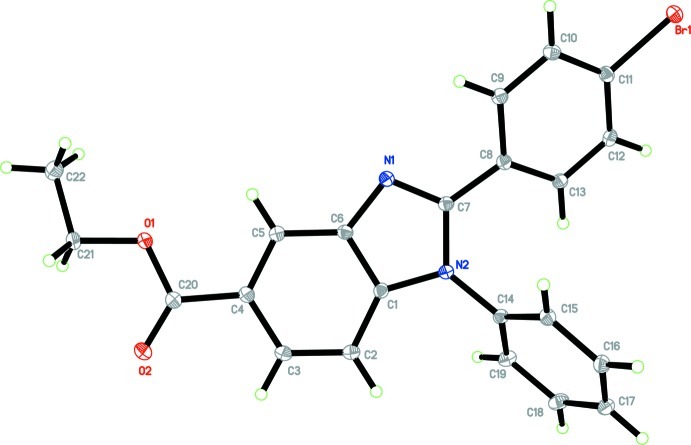
The molecular structure of the title compound, showing 30% probability displacement ellipsoids.

**Fig. 2.**
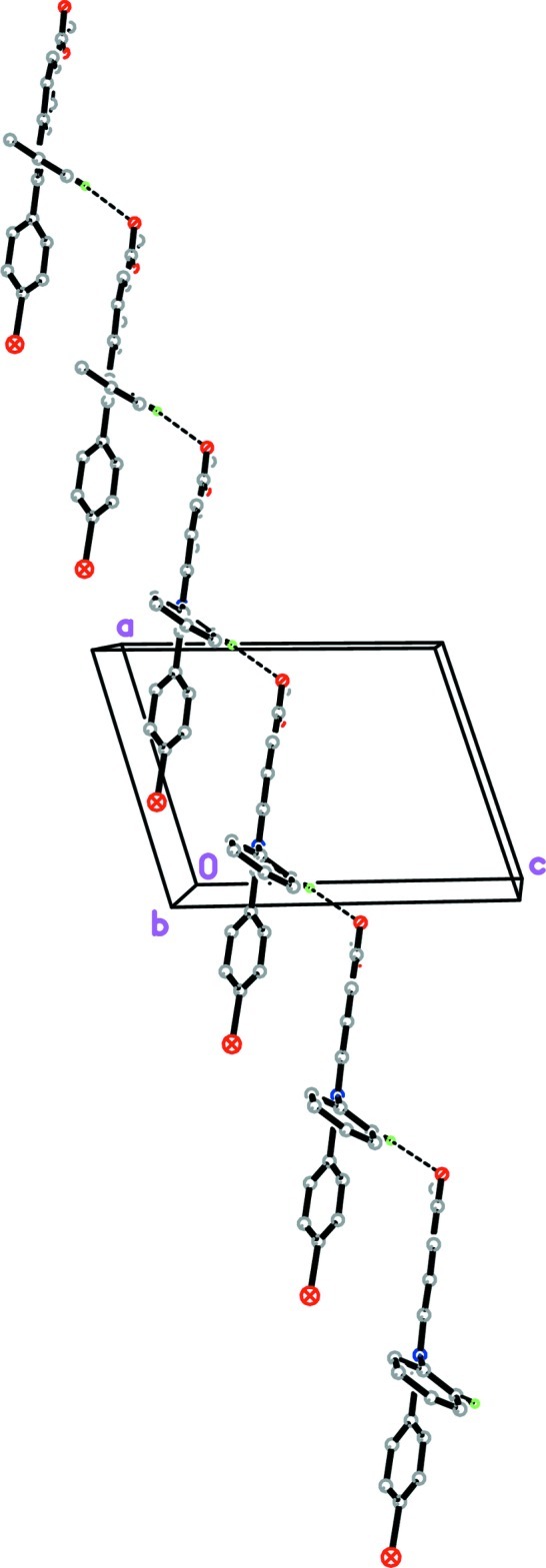
The crystal packing of the title compound. The H atoms not involved in the intermolecular interactions (dashed lines) have been omitted for clarity.

## supplementary crystallographic information

### Comment

Benzimidazoles are a class of bioactive heterocyclic compounds which exhibit a wide range of activities such as anti-cancer (Townsend & Revankar, 1970), anti-HIV (Rao *et al.*, 2002), anti-inflammatory (Thakurdesai *et al.*, 2007) and anthelmintics (Dubey & Sanyal, 2010). The primary mechanism of action of benzimidazoles as anthelmintics is by binding to free β-tubulin and inhibiting its polymerization (Lacey, 1990). A number of benzimidazoles have been shown to also inhibit mammalian tubulin polymerization and to be aneugenic *in vivo*.

The molecular structure is shown in Fig. 1. Bond lengths and angles are within normal ranges and are comparable to a related structure (Arumugam *et al.*,2010). The benzimidazole ring system (N1/N2/C1—C7) is essentially planar with a maximum deviation of 0.017 (1) Å for atom C7. The dihedral angles of the benzimidazole ring (N1/N2/C1—C7) with the phenyl ring (C14–C19) and the bromo-substituted benzene ring (C8–C13) are 27.79 (6) and 64.43 (6)°, respectively.

In the crystal packing (Fig. 2), intermolecular C15—H15A···O2^i^ (Table 1) hydrogen bonds link the molecules into one-dimensional zigzag chains along the *a*-axis. In addition, the crystal structure is further stabilized by the intermolecular C21—H21A···*Cg*1^iii^ and C19—H19A···*Cg*2^ii^ (Table 1) interactions (*Cg*1 and *Cg*2 are the centroids of N1/N1/C1/C6/C7 and C8–C13 rings, respectively).

### Experimental

Ethyl 3-amino-4-(phenyl amino) benzoate (0.84 mmol) and sodium metabisulfite adduct of bromo benzaldehyde (1.68 mmol) were dissolved in DMF. The reaction mixture was reflux at 403K for 2 h. After completion, the reaction mixture was diluted in ethyl acetate (20 ml) and washed with water (20 ml). The organic layer was collected, dried over Na_2_SO_4_ and the evaporated *in vacuo* to yield the product. The product was recrystallized from ethyl acetate.

### Refinement

All H atoms were positioned geometrically [C–H = 0.95–0.99 Å] and refined using a riding model with *U*_iso_(H) = 1.2 and 1.5 *U*_eq_(C). A rotating group model was applied to the methyl group.

### Crystal data

**Table:**

C_22_H_17_BrN_2_O_2_	*Z* = 2
*M**_r_* = 421.29	*F*(000) = 428
Triclinic, *P*1	*D*_x_ = 1.528 Mg m^−^^3^
Hall symbol: -P 1	Mo *K*α radiation, λ = 0.71073 Å
*a* = 9.3121 (2) Å	Cell parameters from 9874 reflections
*b* = 9.8136 (2) Å	θ = 2.4–32.7°
*c* = 11.8458 (2) Å	µ = 2.27 mm^−^^1^
α = 108.217 (1)°	*T* = 100 K
β = 101.135 (1)°	Block, colourless
γ = 109.361 (1)°	0.42 × 0.33 × 0.15 mm
*V* = 915.39 (3) Å^3^	

### Data collection

**Table:**

Bruker SMART APEXII CCD area-detector diffractometer	5326 independent reflections
Radiation source: fine-focus sealed tube	4846 reflections with *I* > *I* > 2σ(*I*)
Graphite monochromator	*R*_int_ = 0.025
φ and ω scans	θ_max_ = 30.0°, θ_min_ = 1.9°
Absorption correction: multi-scan (*SADABS*; Bruker, 2009)	*h* = −13→13
*T*_min_ = 0.450, *T*_max_ = 0.728	*k* = −13→13
21718 measured reflections	*l* = −16→16

### Refinement

**Table:**

Refinement on *F*^2^	Primary atom site location: structure-invariant direct methods
Least-squares matrix: full	Secondary atom site location: difference Fourier map
*R*[*F*^2^ > 2σ(*F*^2^)] = 0.027	Hydrogen site location: inferred from neighbouring sites
*wR*(*F*^2^) = 0.071	H-atom parameters constrained
*S* = 1.06	*w* = 1/[σ^2^(*F*_o_^2^) + (0.0366*P*)^2^ + 0.3723*P*] where *P* = (*F*_o_^2^ + 2*F*_c_^2^)/3
5326 reflections	(Δ/σ)_max_ = 0.001
245 parameters	Δρ_max_ = 0.53 e Å^−^^3^
0 restraints	Δρ_min_ = −0.65 e Å^−^^3^

### Special details

**Table:**

Experimental. The crystal was placed in the cold stream of an Oxford Cryosystems Cobra open-flow nitrogen cryostat (Cosier & Glazer, 1986) operating at 100.0 (1) K.
Geometry. All e.s.d.'s (except the e.s.d. in the dihedral angle between two l.s. planes) are estimated using the full covariance matrix. The cell e.s.d.'s are taken into account individually in the estimation of e.s.d.'s in distances, angles and torsion angles; correlations between e.s.d.'s in cell parameters are only used when they are defined by crystal symmetry. An approximate (isotropic) treatment of cell e.s.d.'s is used for estimating e.s.d.'s involving l.s. planes.
Refinement. Refinement of *F*^2^ against ALL reflections. The weighted *R*-factor *wR* and goodness of fit *S* are based on *F*^2^, conventional *R*-factors *R* are based on *F*, with *F* set to zero for negative *F*^2^. The threshold expression of *F*^2^ > σ(*F*^2^) is used only for calculating *R*-factors(gt) *etc*. and is not relevant to the choice of reflections for refinement. *R*-factors based on *F*^2^ are statistically about twice as large as those based on *F*, and *R*- factors based on ALL data will be even larger.

### Fractional atomic coordinates and isotropic or equivalent isotropic displacement parameters (Å^2^)

**Table:**

	*x*	*y*	*z*	*U*_iso_*/*U*_eq_	
Br1	−0.680059 (17)	−0.321236 (17)	−0.071893 (14)	0.02360 (5)	
O1	0.66395 (12)	−0.10601 (12)	0.40079 (10)	0.0201 (2)	
O2	0.84619 (13)	0.14498 (13)	0.46778 (10)	0.0230 (2)	
N1	0.12223 (14)	−0.10878 (14)	0.20976 (11)	0.0180 (2)	
N2	0.16438 (14)	0.14058 (14)	0.23396 (11)	0.0163 (2)	
C1	0.31426 (17)	0.14481 (16)	0.28300 (13)	0.0167 (2)	
C2	0.46861 (18)	0.26876 (17)	0.34068 (14)	0.0202 (3)	
H2A	0.4867	0.3736	0.3505	0.024*	
C3	0.59380 (18)	0.23123 (17)	0.38280 (14)	0.0205 (3)	
H3A	0.7005	0.3123	0.4226	0.025*	
C4	0.56651 (17)	0.07511 (17)	0.36785 (13)	0.0181 (3)	
C5	0.41259 (17)	−0.04732 (17)	0.30997 (13)	0.0184 (3)	
H5A	0.3946	−0.1522	0.2997	0.022*	
C6	0.28543 (17)	−0.01088 (16)	0.26745 (13)	0.0168 (2)	
C7	0.05354 (17)	−0.01604 (16)	0.19171 (12)	0.0160 (2)	
C8	−0.12145 (16)	−0.07748 (16)	0.13144 (12)	0.0164 (2)	
C9	−0.21833 (17)	−0.21394 (17)	0.14191 (13)	0.0182 (3)	
H9A	−0.1695	−0.2591	0.1889	0.022*	
C10	−0.38444 (17)	−0.28386 (17)	0.08468 (13)	0.0192 (3)	
H10A	−0.4497	−0.3741	0.0945	0.023*	
C11	−0.45403 (17)	−0.21985 (17)	0.01266 (13)	0.0183 (3)	
C12	−0.36095 (17)	−0.08657 (17)	−0.00143 (13)	0.0183 (3)	
H12A	−0.4102	−0.0453	−0.0520	0.022*	
C13	−0.19447 (17)	−0.01399 (16)	0.05949 (13)	0.0172 (2)	
H13A	−0.1303	0.0788	0.0522	0.021*	
C14	0.13582 (17)	0.27765 (16)	0.24107 (13)	0.0168 (2)	
C15	0.04564 (17)	0.32281 (17)	0.31392 (13)	0.0187 (3)	
H15A	0.0000	0.2619	0.3569	0.022*	
C16	0.02336 (18)	0.45862 (18)	0.32284 (14)	0.0220 (3)	
H16A	−0.0369	0.4914	0.3732	0.026*	
C17	0.08845 (19)	0.54698 (18)	0.25870 (15)	0.0236 (3)	
H17A	0.0729	0.6397	0.2654	0.028*	
C18	0.1760 (2)	0.49909 (18)	0.18500 (15)	0.0250 (3)	
H18A	0.2188	0.5582	0.1400	0.030*	
C19	0.20176 (19)	0.36444 (17)	0.17651 (14)	0.0208 (3)	
H19A	0.2635	0.3326	0.1273	0.025*	
C20	0.70792 (17)	0.04581 (17)	0.41792 (13)	0.0183 (3)	
C21	0.79333 (17)	−0.14750 (17)	0.44476 (13)	0.0199 (3)	
H21A	0.8430	−0.0922	0.5375	0.024*	
H21B	0.8778	−0.1179	0.4063	0.024*	
C22	0.7174 (2)	−0.32313 (19)	0.40531 (15)	0.0264 (3)	
H22A	0.8004	−0.3580	0.4316	0.040*	
H22B	0.6670	−0.3758	0.3135	0.040*	
H22C	0.6353	−0.3502	0.4451	0.040*	

### Atomic displacement parameters (Å^2^)

**Table:**

	*U*^11^	*U*^22^	*U*^33^	*U*^12^	*U*^13^	*U*^23^
Br1	0.01607 (8)	0.02227 (8)	0.02755 (8)	0.00650 (6)	0.00244 (6)	0.00860 (6)
O1	0.0153 (5)	0.0192 (5)	0.0243 (5)	0.0073 (4)	0.0042 (4)	0.0083 (4)
O2	0.0168 (5)	0.0232 (5)	0.0259 (5)	0.0062 (4)	0.0054 (4)	0.0095 (4)
N1	0.0162 (5)	0.0167 (5)	0.0189 (5)	0.0060 (4)	0.0031 (4)	0.0071 (5)
N2	0.0162 (5)	0.0144 (5)	0.0182 (5)	0.0060 (4)	0.0053 (4)	0.0071 (4)
C1	0.0171 (6)	0.0170 (6)	0.0176 (6)	0.0076 (5)	0.0063 (5)	0.0082 (5)
C2	0.0196 (7)	0.0163 (6)	0.0232 (7)	0.0057 (5)	0.0067 (5)	0.0083 (5)
C3	0.0167 (6)	0.0182 (6)	0.0235 (7)	0.0042 (5)	0.0061 (5)	0.0082 (5)
C4	0.0167 (6)	0.0190 (6)	0.0182 (6)	0.0073 (5)	0.0059 (5)	0.0075 (5)
C5	0.0183 (6)	0.0171 (6)	0.0193 (6)	0.0071 (5)	0.0059 (5)	0.0073 (5)
C6	0.0166 (6)	0.0155 (6)	0.0160 (6)	0.0052 (5)	0.0042 (5)	0.0060 (5)
C7	0.0173 (6)	0.0148 (6)	0.0147 (6)	0.0057 (5)	0.0047 (5)	0.0061 (5)
C8	0.0158 (6)	0.0168 (6)	0.0141 (6)	0.0063 (5)	0.0039 (5)	0.0046 (5)
C9	0.0195 (6)	0.0185 (6)	0.0158 (6)	0.0077 (5)	0.0044 (5)	0.0071 (5)
C10	0.0191 (6)	0.0179 (6)	0.0175 (6)	0.0053 (5)	0.0052 (5)	0.0063 (5)
C11	0.0164 (6)	0.0193 (6)	0.0170 (6)	0.0077 (5)	0.0049 (5)	0.0049 (5)
C12	0.0207 (7)	0.0188 (6)	0.0166 (6)	0.0111 (5)	0.0053 (5)	0.0062 (5)
C13	0.0188 (6)	0.0164 (6)	0.0166 (6)	0.0077 (5)	0.0064 (5)	0.0064 (5)
C14	0.0171 (6)	0.0151 (6)	0.0163 (6)	0.0061 (5)	0.0035 (5)	0.0058 (5)
C15	0.0176 (6)	0.0197 (6)	0.0184 (6)	0.0075 (5)	0.0055 (5)	0.0079 (5)
C16	0.0202 (7)	0.0218 (7)	0.0230 (7)	0.0107 (6)	0.0062 (5)	0.0064 (6)
C17	0.0234 (7)	0.0170 (6)	0.0267 (7)	0.0084 (6)	0.0027 (6)	0.0076 (6)
C18	0.0322 (8)	0.0193 (7)	0.0236 (7)	0.0087 (6)	0.0086 (6)	0.0117 (6)
C19	0.0262 (7)	0.0179 (6)	0.0190 (6)	0.0087 (6)	0.0100 (6)	0.0075 (5)
C20	0.0183 (6)	0.0200 (6)	0.0172 (6)	0.0080 (5)	0.0074 (5)	0.0076 (5)
C21	0.0178 (6)	0.0230 (7)	0.0187 (6)	0.0105 (6)	0.0043 (5)	0.0075 (5)
C22	0.0304 (8)	0.0243 (7)	0.0254 (7)	0.0135 (6)	0.0076 (6)	0.0100 (6)

### Geometric parameters (Å, º)

**Table:**

Br1—C11	1.8967 (14)	C10—C11	1.391 (2)
O1—C20	1.3428 (17)	C10—H10A	0.9500
O1—C21	1.4506 (17)	C11—C12	1.390 (2)
O2—C20	1.2124 (18)	C12—C13	1.3949 (19)
N1—C7	1.3182 (17)	C12—H12A	0.9500
N1—C6	1.3856 (18)	C13—H13A	0.9500
N2—C1	1.3858 (17)	C14—C19	1.3889 (19)
N2—C7	1.3950 (17)	C14—C15	1.3906 (19)
N2—C14	1.4360 (17)	C15—C16	1.391 (2)
C1—C2	1.398 (2)	C15—H15A	0.9500
C1—C6	1.4047 (19)	C16—C17	1.392 (2)
C2—C3	1.385 (2)	C16—H16A	0.9500
C2—H2A	0.9500	C17—C18	1.386 (2)
C3—C4	1.414 (2)	C17—H17A	0.9500
C3—H3A	0.9500	C18—C19	1.396 (2)
C4—C5	1.3898 (19)	C18—H18A	0.9500
C4—C20	1.4915 (19)	C19—H19A	0.9500
C5—C6	1.3953 (19)	C21—C22	1.500 (2)
C5—H5A	0.9500	C21—H21A	0.9900
C7—C8	1.4692 (19)	C21—H21B	0.9900
C8—C13	1.4022 (19)	C22—H22A	0.9800
C8—C9	1.4031 (19)	C22—H22B	0.9800
C9—C10	1.387 (2)	C22—H22C	0.9800
C9—H9A	0.9500		
			
C20—O1—C21	115.96 (11)	C11—C12—H12A	120.3
C7—N1—C6	105.29 (11)	C13—C12—H12A	120.3
C1—N2—C7	106.01 (11)	C12—C13—C8	120.27 (13)
C1—N2—C14	124.37 (11)	C12—C13—H13A	119.9
C7—N2—C14	129.28 (12)	C8—C13—H13A	119.9
N2—C1—C2	131.97 (13)	C19—C14—C15	121.23 (13)
N2—C1—C6	105.65 (12)	C19—C14—N2	119.07 (12)
C2—C1—C6	122.36 (13)	C15—C14—N2	119.69 (12)
C3—C2—C1	116.72 (13)	C14—C15—C16	118.92 (13)
C3—C2—H2A	121.6	C14—C15—H15A	120.5
C1—C2—H2A	121.6	C16—C15—H15A	120.5
C2—C3—C4	121.52 (13)	C15—C16—C17	120.63 (14)
C2—C3—H3A	119.2	C15—C16—H16A	119.7
C4—C3—H3A	119.2	C17—C16—H16A	119.7
C5—C4—C3	121.27 (13)	C18—C17—C16	119.74 (14)
C5—C4—C20	120.79 (13)	C18—C17—H17A	120.1
C3—C4—C20	117.94 (13)	C16—C17—H17A	120.1
C4—C5—C6	117.74 (13)	C17—C18—C19	120.41 (14)
C4—C5—H5A	121.1	C17—C18—H18A	119.8
C6—C5—H5A	121.1	C19—C18—H18A	119.8
N1—C6—C5	129.29 (13)	C14—C19—C18	119.06 (13)
N1—C6—C1	110.30 (12)	C14—C19—H19A	120.5
C5—C6—C1	120.40 (13)	C18—C19—H19A	120.5
N1—C7—N2	112.74 (12)	O2—C20—O1	123.35 (13)
N1—C7—C8	121.69 (12)	O2—C20—C4	125.07 (13)
N2—C7—C8	125.56 (12)	O1—C20—C4	111.58 (12)
C13—C8—C9	119.05 (13)	O1—C21—C22	106.03 (12)
C13—C8—C7	124.21 (12)	O1—C21—H21A	110.5
C9—C8—C7	116.64 (12)	C22—C21—H21A	110.5
C10—C9—C8	120.93 (13)	O1—C21—H21B	110.5
C10—C9—H9A	119.5	C22—C21—H21B	110.5
C8—C9—H9A	119.5	H21A—C21—H21B	108.7
C9—C10—C11	119.00 (13)	C21—C22—H22A	109.5
C9—C10—H10A	120.5	C21—C22—H22B	109.5
C11—C10—H10A	120.5	H22A—C22—H22B	109.5
C12—C11—C10	121.39 (13)	C21—C22—H22C	109.5
C12—C11—Br1	119.62 (11)	H22A—C22—H22C	109.5
C10—C11—Br1	118.97 (11)	H22B—C22—H22C	109.5
C11—C12—C13	119.32 (13)		
			
C7—N2—C1—C2	−178.43 (15)	C13—C8—C9—C10	−1.5 (2)
C14—N2—C1—C2	−4.5 (2)	C7—C8—C9—C10	−177.86 (12)
C7—N2—C1—C6	0.05 (14)	C8—C9—C10—C11	2.2 (2)
C14—N2—C1—C6	173.96 (12)	C9—C10—C11—C12	−0.9 (2)
N2—C1—C2—C3	178.10 (14)	C9—C10—C11—Br1	177.12 (10)
C6—C1—C2—C3	−0.2 (2)	C10—C11—C12—C13	−1.0 (2)
C1—C2—C3—C4	0.1 (2)	Br1—C11—C12—C13	−179.04 (10)
C2—C3—C4—C5	0.1 (2)	C11—C12—C13—C8	1.7 (2)
C2—C3—C4—C20	−179.00 (13)	C9—C8—C13—C12	−0.5 (2)
C3—C4—C5—C6	−0.3 (2)	C7—C8—C13—C12	175.61 (13)
C20—C4—C5—C6	178.81 (12)	C1—N2—C14—C19	65.95 (18)
C7—N1—C6—C5	178.30 (14)	C7—N2—C14—C19	−121.62 (16)
C7—N1—C6—C1	−0.54 (15)	C1—N2—C14—C15	−112.87 (15)
C4—C5—C6—N1	−178.52 (13)	C7—N2—C14—C15	59.56 (19)
C4—C5—C6—C1	0.2 (2)	C19—C14—C15—C16	−0.8 (2)
N2—C1—C6—N1	0.30 (15)	N2—C14—C15—C16	178.04 (13)
C2—C1—C6—N1	178.95 (13)	C14—C15—C16—C17	0.8 (2)
N2—C1—C6—C5	−178.66 (12)	C15—C16—C17—C18	0.1 (2)
C2—C1—C6—C5	0.0 (2)	C16—C17—C18—C19	−1.1 (2)
C6—N1—C7—N2	0.58 (15)	C15—C14—C19—C18	−0.2 (2)
C6—N1—C7—C8	179.77 (12)	N2—C14—C19—C18	−179.03 (13)
C1—N2—C7—N1	−0.40 (15)	C17—C18—C19—C14	1.2 (2)
C14—N2—C7—N1	−173.91 (13)	C21—O1—C20—O2	−0.62 (19)
C1—N2—C7—C8	−179.56 (12)	C21—O1—C20—C4	179.17 (11)
C14—N2—C7—C8	6.9 (2)	C5—C4—C20—O2	178.78 (14)
N1—C7—C8—C13	−149.98 (14)	C3—C4—C20—O2	−2.1 (2)
N2—C7—C8—C13	29.1 (2)	C5—C4—C20—O1	−1.01 (18)
N1—C7—C8—C9	26.20 (19)	C3—C4—C20—O1	178.09 (12)
N2—C7—C8—C9	−154.71 (13)	C20—O1—C21—C22	−175.08 (12)

### Hydrogen-bond geometry (Å, º)

Cg1 and Cg2 are the centroids of the N1/N1/C1/C6/C7 and C8–C13 rings, respectively.

**Table:**

*D*—H···*A*	*D*—H	H···*A*	*D*···*A*	*D*—H···*A*
C15—H15*A*···O2^i^	0.95	2.38	3.311 (2)	166
C19—H19*A*···*Cg*2^ii^	0.95	2.59	3.4534 (18)	152
C21—H21*A*···*Cg*1^iii^	0.99	2.64	3.5288 (15)	149

Symmetry codes: (i) *x*−1, *y*, *z*; (ii) −*x*, −*y*, −*z*; (iii) −*x*+1, −*y*, −*z*+1.
